# Emotion Processes Predicting Outbursts and Functional Impact in Misophonia

**DOI:** 10.3389/fpsyg.2022.903142

**Published:** 2022-07-04

**Authors:** Qiaochu Wang, Silia Vitoratou, Nora Uglik-Marucha, Jane Gregory

**Affiliations:** ^1^Psychometrics and Measurement Lab, Biostatistics and Health Informatics Department, Institute of Psychiatry, Psychology and Neuroscience, King’s College London, London, United Kingdom; ^2^Department of Experimental Psychology, University of Oxford, Oxford, United Kingdom; ^3^Oxford Health Specialist Psychological Interventions Centre, Oxford Health NHS Foundation Trust, Oxford, United Kingdom

**Keywords:** misophonia, S-Five, misophonic outbursts, misophonic impact, disgust sensitivity, emotion processes

## Abstract

Misophonia involves a decreased tolerance to certain sounds and is associated with a range of emotions and emotion processes. In addition to the distress caused by misophonia, some individuals report having aggressive outbursts and significant impact on doing things they would like to be able to do. This study aimed to examine whether misophonia-specific cognitive and emotional processes were associated with misophonic outbursts and impact, and whether these relationships could be explained in part by emotion processes not specific to misophonia. A sample of 703 individuals, 315 of whom identified with having misophonia, completed measures of misophonia, depression and anxiety symptoms, anxiety and disgust sensitivity, interoception and beliefs about emotions. Exploratory correlation and regression analyses were used to build mediation models, which were tested using multiple linear regression. Externalising appraisals (blaming others for causing one’s reaction to sounds) were positively associated with misophonic outbursts, and this relationship was partially explained by anxiety symptoms and disgust sensitivity. Sense of emotional threat in misophonia predicted functional impact of misophonia, and this was partially explained by depression symptoms and negative beliefs about emotions. Anxiety sensitivity and interoception were not significant independent predictors of misophonic outbursts or functional impact. These results provide support for the relevance of emotion processes in misophonia and highlight the importance of using multi-dimensional measures of misophonia to improve our understanding of the condition.

## Introduction

Misophonia is characterised by decreased tolerance to select sounds that might be only mildly aversive to others, which can lead to intense emotional, physical and behavioural reactions and functional impairment (Swedo et al., 2021). Current research suggests that its aetiology is complex, possibly influenced by individual perception, past experiences, context, acoustic features of sounds (Jastreboff and Jastreboff, 2002; Bernstein et al., 2013; Rouw and Erfanian, 2018) and atypical connectivity in the brain (Kumar et al., 2017). In addition to the distress caused by misophonia, there are negative outcomes in terms of aggressive outbursts and the impact of the disorder on being able to live a full and productive life (Swedo et al., 2021; Vitoratou et al., 2021).

One study found that although patients frequently reported fears about resorting to violence, physical outbursts in misophonia were rare (Jager et al., 2020). This was supported by the psychometric analysis of the S-Five, a measure of misophonia severity, in a sample of individuals identifying with the condition (Vitoratou et al., 2021). Within the “Outburst” factor, mean scores were approximately 5 (out of a possible 10) on items about shouting, verbal aggression and worries about acting on violent thoughts, and lower for physical aggression (mean 2.7 out of 10) and violence (2.3).

It is not clear what factors contribute to an individual’s tendency to have verbal or physical outbursts in misophonia. Anger is one of the most frequently reported emotions in response to trigger sounds (Schröder et al., 2013; Vitoratou et al., 2021), but the frequency of anger reactions to triggers has only a low correlation with outbursts (Vitoratou et al., 2021). Two studies examined the relationship between misophonia severity and general anger outbursts (i.e., not misophonia-specific), and found that the relationship was partially mediated by the presence of anxiety symptoms (Wu et al., 2014; Zhou et al., 2017).

Another study found that anxiety sensitivity, a relatively stable, transdiagnostic trait related to fearful beliefs about anxiety symptoms (Hovenkamp-Hermelink et al., 2019), strengthened the relationship between misophonia symptoms and aggression (Schadegg et al., 2021). That is, at higher levels of anxiety sensitivity (more fear of anxiety sensations), misophonia was more strongly associated with aggression. However, this study did not control for anxiety symptoms, which are also associated with anxiety sensitivity (Wheaton et al., 2012). It is therefore not clear whether it is current symptoms of anxiety or the trait of anxiety sensitivity, or both, that influences the relationship between misophonia and general aggressive outbursts.

The studies described above did not examine aggression in the context of misophonia-specific outbursts, focusing instead on general aggression and outbursts. Barahmand et al. (2021) examined traits of dealing with emotions as predictors of misophonic behaviour, which included misophonia-specific aggression. They found that disgust sensitivity was associated with misophonic behaviours, but this relationship was fully mediated by emotion dysregulation. They proposed that misophonic behaviours emerge from an interplay of emotions and cognitive processes.

Misophonia-specific outbursts are captured in one of the dimensions of the S-Five scale (Vitoratou et al., 2021). In the initial psychometric analysis of this scale, outbursts were positively associated with all the other dimensions of the scale: internalising and externalising appraisals, sense of emotional threat and functional impact. Given that aggression is associated with other-directed blame (Kulik and Brown, 1979), the association between misophonic outbursts and externalising appraisals is worth exploring further. Individuals with misophonia have expressed negative assumptions about the character of those making unpleasant sounds (Edelstein et al., 2013; Rouw and Erfanian, 2018; Vitoratou et al., 2021), as captured in the externalising factor, but it is not yet clear whether there is a direct or indirect relationship between these interpretations in the moment and aggressive outbursts.

Outbursts in misophonia were also associated with symptoms of depression and anxiety, and functional and social impairment (Vitoratou et al., 2021). Further research into predictors of misophonia-specific outbursts would be helpful for identifying potential targets to address this negative outcome for individuals with misophonia.

Beyond outbursts, individuals living with misophonia also report impact on social and occupational functioning (Edelstein et al., 2013; Rouw and Erfanian, 2018), loss of enjoyment (Hocaoglu, 2018), not being able to go places and see the people they would like to see and concern about future impact of the condition (Vitoratou et al., 2021). In a study of 828 individuals with self-identified misophonia, an average score of 45 out of a possible 50 was found for the S-Five variable described as emotional threat, which captures a sense of misophonia-specific emotional dysregulation, with items about feeling distressed, trapped and helpless if unable to get away from sounds (Vitoratou et al., 2021). If day-to-day sounds like eating and breathing can potentially lead to such intense reactions, it is not surprising that individuals with misophonia would report limited lives and concerns about future opportunities. As yet, it is not clear which psychological processes may contribute to greater impact of misophonia, in terms of the perceived limitations of misophonia on daily functioning and concerns about future functioning. To our knowledge, there is no prior research examining this specifically.

### Summary and Aims of the Study

Existing research has shown us that symptoms of misophonia are likely associated with a range of emotion processes. Based on the literature, it seems that there are several potential emotion processes associated with misophonia, including an increased propensity to experience certain emotions (Barahmand et al., 2021), awareness of bodily sensations connected to emotions (interoception; Kumar et al., 2017; McKay et al., 2018), beliefs about the nature and consequences of sensations caused by emotion (e.g., anxiety sensitivity; Schadegg et al., 2021) and the presence of symptoms of disorders related to emotional health, such as anxiety and depression (Erfanian et al., 2019; Jager et al., 2020).

Further investigation is needed to improve our understanding of the role of these emotion processes in misophonia. We also need research to distinguish between misophonia-specific processes (e.g., a sense of uncontrollable emotions in the presence of sounds) and general processes (e.g., believing that one’s emotions are uncontrollable, in general).

The aim of this exploratory study was to examine these processes in a sample of both individuals who identify with having misophonia and individuals who do not identify with having misophonia. We aimed to examine this specifically in relation to two negative outcomes in misophonia: aggressive outbursts or fear of having outbursts (henceforth “outbursts”) and the perceived impact in terms of limitations on functioning (henceforth “functional impact”). We theorised that misophonia-specific variables would be significant predictors of the outcome variables and aimed to investigate whether these relationships could be partially or fully explained by variables related to emotions and general emotion processing.

Specifically, we hypothesised misophonic outbursts would be predicted by the S-Five variable of externalising appraisals, that is, the tendency to put blame for one’s reactions to sounds on to the person making the sound. We also hypothesised that functional impact would be predicted by the S-Five variable of emotional threat.

Drawing from the existing misophonia literature, the additional predictor variables we examined were interoception, disgust propensity and sensitivity, anxiety sensitivity, beliefs about emotions and symptoms of depression and anxiety. As there were no previous studies examining these variables in the context of misophonia-specific outcomes at the time of designing the research, we did not form any *a priori* hypotheses regarding their possible impact. Instead, we aimed to develop models to be tested in an exploratory study.

## Materials and Methods

### Recruitment

As part of a wider project studying misophonia, participants were recruited from two different sources. The sampling service Prolific was used to recruit participants from the general population. To ensure our sample also included a large number of individuals experiencing symptoms of misophonia, we also recruited from misophonia support groups on social media (e.g., Facebook). Inclusion criteria included being older than 18 years old, with enough fluency in English to complete the questionnaire, and without any severe learning disabilities. Attention-check items were included in the survey and participants were removed if they did not complete these satisfactorily. All participants gave informed consent before beginning the survey.

### Measures

Information on the participants’ age, gender, ethnicity, education and occupation were collected. During this process, participants were also asked whether they self-identify with having misophonia (yes, no or unsure). Participants were then presented with the list of measures listed below.

#### Selective Sound Sensitivity Syndrome Scale

The S-Five is a multidimensional tool for measuring misophonia severity (Vitoratou et al., 2021). It has five distinct factors: internalising and externalising appraisals, emotional threat, outbursts and functional impact. The five factors demonstrated satisfactory internal consistency (*α* ≥ 0.83) and stability in time (stability coefficients >0.80 in all items and factors; Vitoratou et al., 2021). The supplementary trigger checklist of the S-Five was not used in the present study.

#### Anxiety Sensitivity Index-3

The ASI-3 (Taylor et al., 2007) is an 18-item inventory that measures anxiety sensitivity, the fear of sensations related to anxiety, across three dimensions: physical, cognitive and social concerns. All three subscales have demonstrated high reliability in internal consistency in both clinical and non-clinical samples (*α* > 0.70).

#### Body Consciousness Questionnaire

The BCQ (Miller et al., 1981) is a 15-item self-report questionnaire measuring three components: public body consciousness, body competence and private body consciousness. Participants completed the whole scale, but only the private body consciousness subscale (*α* = 0.69) was used in this study for the purpose of measuring interoception, one’s awareness of internal bodily sensations. This subscale was found to be higher in those with misophonia than without (Kumar et al., 2017), and there was no current theoretical rationale for inclusion of the other two subscales. All subscales have demonstrated high test–retest reliability.

#### Disgust Propensity and Sensitivity Scale-Revised

We used the 12-item, shortened version of the DPSS-R (Fergus and Valentiner, 2009), the full version of which contains 16-item (van Overveld et al., 2006). The items are measured on a five-point scale (from never to always) and form two factors. Disgust propensity is the ease of which one is disgusted, whilst disgust sensitivity is how bothered an individual is by their disgust (van Overveld et al., 2006). Both reduced-item subscales show good internal consistency, at *α* = 0.83 for disgust propensity and *α* = 0.80 for disgust sensitivity and share a moderate to strong correlation (*r* = 0.59) with one another (Fergus and Valentiner, 2009). In the present study, we used the combined propensity and sensitivity, henceforth referred to as disgust sensitivity.

#### Generalised Anxiety Disorder-7

The GAD7 is a widely used, valid and reliable scale measuring symptoms of anxiety (Spitzer et al., 2006). It has seven items, each measuring the frequency of symptoms (from *not at all* to *nearly every day*).

#### Leahy Emotional Sensitivity Scale-II

The Leahy Emotional Sensitivity Scale-II (Leahy, 2012; Unpublished Manuscript)^1^ consists of 28 items measuring 14 dimensions, with a six-point ordinal response scale measuring negative beliefs about emotions (e.g., “I feel ashamed of my feelings”), with higher scores indicating more negatively held beliefs.

#### Patient Health Questionnaire-9

The PHQ9 is a widely used measure of symptoms of depression (Kroenke et al., 2001). It has nine items, each measuring the frequency of symptoms (from *not at all* to *nearly every day*) and has good validity and reliability.

### Data Analysis

In this work, we focused on two main models. First, we studied the effect of the externalising factor on outbursts, followed by the effect of the threat factor on functional impact. In both models, we adjusted for age, gender, identifying as having misophonia or not and a set of covariates. The set of covariates was identified in the literature and was verified first by exploring the inter-correlations between the measures used in the study. That is, pairwise correlations of the key variables of interest (interoception, anxiety sensitivity, disgust sensitivity, beliefs about emotions, anxiety symptoms, depression symptoms, as well as the S-Five total score and factors, along with age and gender) were examined separately for those who identified with and without misophonia. Variables that did not show significant correlations were removed from consideration.

Next, forward stepwise linear regression was conducted to build comprehensive models. At each step, all possible combinations of predictor variables were entered, and any variables found to be non-significant at any step in this process were removed. We chose a stepwise selection procedure to account for possible multicollinearity between predictor variables. Significant variables were carried forwards to the next step, increasing the number of variables in the model until the best fitting model was found. This process yielded the models with the combinations of variables that explained the largest amount of the outcome’s variability, as determined by the model’s *R*^2^ value, the percentage of variability explained by the included variables. The resulting models were carried forwards into mediation analyses.

Lastly, parallel mediation analyses were carried out with the PROCESS macro (Hayes, 2017), which uses the theoretical Sobel test and Baron and Kenny’s four steps to determine mediation (Baron and Kenny, 1986), as well as a bootstrapping procedure to test the hypothesis. Unstandardised indirect effects were computed for each of 5,000 bootstrapped samples, and the 95% CI was computed by determining the indirect effects at the 2.5th and 97.5th percentiles. All data analyses were conducted on IBM SPSS Statistics for Windows (Version 26.0. Armonk, NY: IBM Corp).

## Results

A total of 728 participants submitted completed surveys. Of these, 25 were removed for not satisfactorily passing attention check items, leaving a total sample of 703. Within this, 396 were recruited from the sampling service Prolific, and 307 were recruited from misophonia groups on social media. Participants were then split into two groups based on their answer to the question, “Do you identify with having misophonia?” Those who responded yes were labelled as “Identifying with having misophonia” and those who said no, or that they were unsure, were labelled as “Not identifying with having misophonia.” Participant demographics are presented in Table 1 and comparisons between the two groups all variables of interest are presented in Table 2. Comparisons between means are presented for variables with normal distributions and comparisons between medians are presented for skewed variables.

**Table 1:** Demographic characteristics of the study sample.

	Identifying misophonia		Not identifying misophonia		Total	
	*n* = 315		*n* = 388		*N* = 703	
	Median	IQR	Median	IQR	Median	IQR
Age	38.0	25.0	47.50	28.0	43.0	28.0
	**No.**	**%**	**No.**	**%**	**No.**	**%**
Gender						
Female	252	80.0	179	46.1	431	61.3
Male	50	15.9	206	53.1	256	36.4
Non-binary	12	0.6	1	0.3	13	1.8
Other	1	0.3	2	0.5	3	0.4

*IQR, interquartile range*.

**Table 2:** Descriptive indices and comparison between groups.

	Identifying misophonia		Not identifying misophonia		Comparison	
	**Median**	**IQR**	**Median**	**IQR**	**MWU**	***p***
S-Five total	156.00	62.50	36.00	51.00	115403.50	<0.001
Externalising	34.00	20.00	17.00	20.00	93376.50	<0.001
Internalising	29.00	25.50	1.00	8.00	108660.00	<0.001
Threat	48.00	6.50	9.00	19.00	117717.50	<0.001
Outburst	17.00	20.00	2.00	7.00	104245.50	<0.001
Impact	27.00	24.50	1.00	5.00	111617.00	<0.001
Anxiety sensitivity	24.00	21.00	19.00	23.00	60976.00	0.007
Anxiety symptoms	9.00	10.00	3.00	6.00	88348.00	<0.001
Depression symptoms	9.00	10.00	5.00	8.00	80031.00	<0.001
	**Mean**	**SD**	**Mean**	**SD**	***t***	***p***
Interoception	13.07	3.38	12.47	3.68	−2.23	0.026
Disgust sensitivity	32.53	7.73	29.28	7.24	−5.69	<0.001
Beliefs about emotions	3.67	0.73	3.16	0.73	−8.72	<0.001

*IQR, interquartile range; MWU, Mann–Whitney U; and SD, standard deviation*.

The pairwise correlations between the variables of interest are presented in Table 3. Externalising was moderately correlated with the outburst subscale. Similarly, threat was moderately correlated with the functional impact subscale. We therefore retained these main relationships of interest for further exploration of our hypotheses. Interoception was not significantly correlated with any of the four target variables in the sample identifying with misophonia and was therefore not carried forwards into the next stage of analysis. All other variables were positively correlated with our four main variables of interest, and with overall misophonia severity, and were therefore retained for the next stage of analysis.

**Table 3:** Intercorrelations for variables of interest in those identifying (and not) with misophonia.

Variable	Anxiety sens	Interoception	Disgust sens	Anxiety symptoms	Belief emotion	Dep symptoms	S-Five					
							Total	Internal	External	Threat	Outburst	Impact
Anxiety sensitivity		0.330 (0.391)	0.528 (0.580)	0.526 (0.622)	0.503 (0.671)	0.400 (0.581)	0.352 (0.463)	0.363 (0.409)	0.109^ns^ (0.295)	0.203 (0.450)	0.215 (0.358)	0.303 (0.358)
Interoception			0.230 (0.381)	0.198 (0.278)	0.195 (0.215)	0.187 (0.238)	0.151 (0.258)	0.182 (0.236)	0.051^ns^ (0.236)	0.106^ns^ (0.238)	0.089^ns^ (0.176)	0.092^ns^ (0.120)
Disgust sensitivity				0.353 (0.366)	0.258 (0.420)	0.262 (0.357)	0.337 (0.418)	0.259 (0.391)	0.169 (0.279)	0.183 (0.435)	0.227 (0.327)	0.272 (0.321)
Anxiety symptoms					0.514 (0.651)	0.697 (0.807)	0.472 (0.437)	0.386 (0.365)	0.188 (0.259)	0.312 (0.450)	0.298 (0.327)	0.407 (0.334)
Belief emotion						0.471 (0.654)	0.493 (0.477)	0.522 (0.403)	0.209 (0.304)	0.272 (0.469)	0.230 (0.357)	0.440 (0.364)
Depression symptoms							0.461 (0.379)	0.366 (0.315)	0.148 (0.212)	0.323 (0.405)	0.276 (0.314)	0.447 (0.255)
**S-Five**												
Total								0.720 (0.737)	0.574 (0.789)	0.613 (0.875)	0.692 (0.741)	0.766 (0.697)
Internalising									0.203 (0.398)	0.337 (0.649)	0.369 (0.600)	0.470 (0.642)
Externalising										0.269 (0.517)	0.307 (0.494)	0.257 (0.406)
Threat											0.370 (0.617)	0.553 (0.615)
Outbursts												0.350 (0.580)
Impact												

*Values in brackets show correlation coefficients of the group not identifying with misophonia. All values were significant at the 0.01 level (two-tailed), except where indicated. ns, correlation is not significant*.

For all the retained variables, correlations were significant in both groups (i.e., those identifying with misophonia and those who did not). We therefore combined the samples at this stage to increase the power of our remaining analyses. In each subsequent analysis, we first controlled for whether participants identified with having misophonia or not.

### Main Effects Analysis

**Figure 1:**
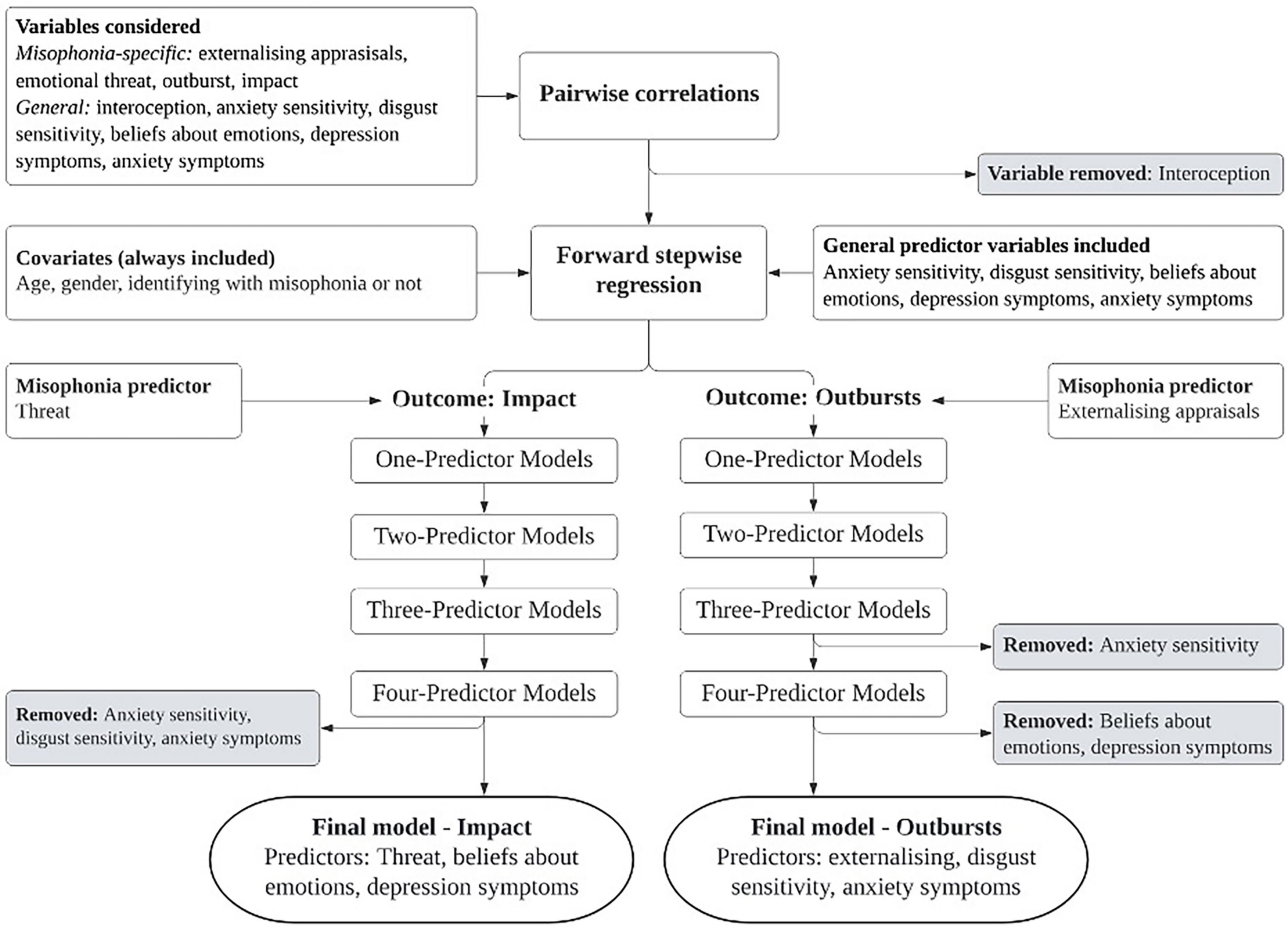
Exploratory process of building models to test in mediation analyses.

In the forward stepwise regression, predictors were added one at a time, and in each model tested, any variables found to be non-significant were removed. Figure 1 summarises the process of building the models to be tested in the mediation analyses, showing the point at which non-significant variables were removed. We present the regression results for only the final models that emerged from this process (see Table 4; full stepwise regression results available on request).

**Table 4:** Regression models for misophonia outcomes outbursts and impact.

Variables	*B*	CI	*t*	*p*	*R* ^2^
Dependent variable: outbursts					**0.461**
(Intercept)	−2.405	[−6.599, 1.790]	−1.126	0.261	
Sex	0.265	[−1.317, 1.847]	0.329	0.743	
Age	−0.049	[−0.096, −0.002]	−2.045	**0.041**	
Misophonia	9.265	[7.514, 11.018]	10.390	**<0.001**	
Disgust Sensitivity	0.125	[0.019, 0.231]	2.309	**0.021**	
Anxiety	0.325	[0.180, 0.469]	4.420	**<0.001**	
Externalising	0.234	[0.177, 0.290]	8.135	**<0.001**	
Dependent variable: functional impact					**0.633**
(Intercept)	−11.114	[−15.471, −6.756]	−5.008	**<0.001**	
Sex	0.258	[−1.357, 1.873]	0.314	0.754	
Age	0.042	[−0.006, 0.089]	1.716	0.087	
Misophonia	8.026	[5.443, 10.609]	6.102	**<0.001**	
Beliefs about emotions	1.934	[0.702, 3.165]	3.084	**0.002**	
Depression	0.312	[0.169, 0.455]	4.283	**<0.001**	
Threat	0.363	[0.292, 0.433]	10.117	**<0.001**	

*Value of *p* in bold highlight the predictors that are statistically significant. Misophonia = identifying with misophonia or not. CI, confidence interval*.

For the dependent variable outbursts, the final model included predictor variables externalising, disgust sensitivity and anxiety symptoms, explaining 46% of the variance. For the dependent variable functional impact, the final model included the predictor variables threat, beliefs about emotions and depression symptoms, explaining 63% of the variance.

### Mediation Analyses

**Figure 2:**
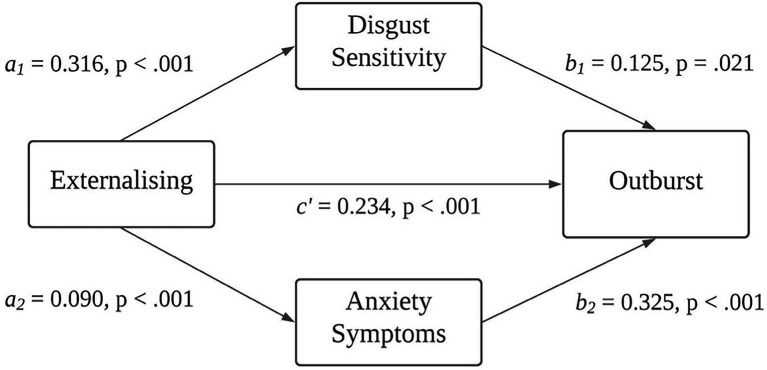
The mediating effect of disgust sensitivity and anxiety symptoms on externalising appraisals and outbursts in misophonia.

After controlling for age, gender and whether they identified with misophonia or not, both disgust sensitivity and anxiety symptoms partially mediated the relationship between externalising and outburst in misophonia (Figure 2). The indirect effect of disgust sensitivity on externalising and outburst was 0.017, and the indirect effect of anxiety was 0.029 (total indirect effect 0.046). The bootstrapped unstandardised indirect effect was 0.046, and the 95% CI ranged from 0.023 to 0.074. Both the indirect and direct effects of externalising on outbursts were statistically significant (*p* < 0.001).

**Figure 3:**
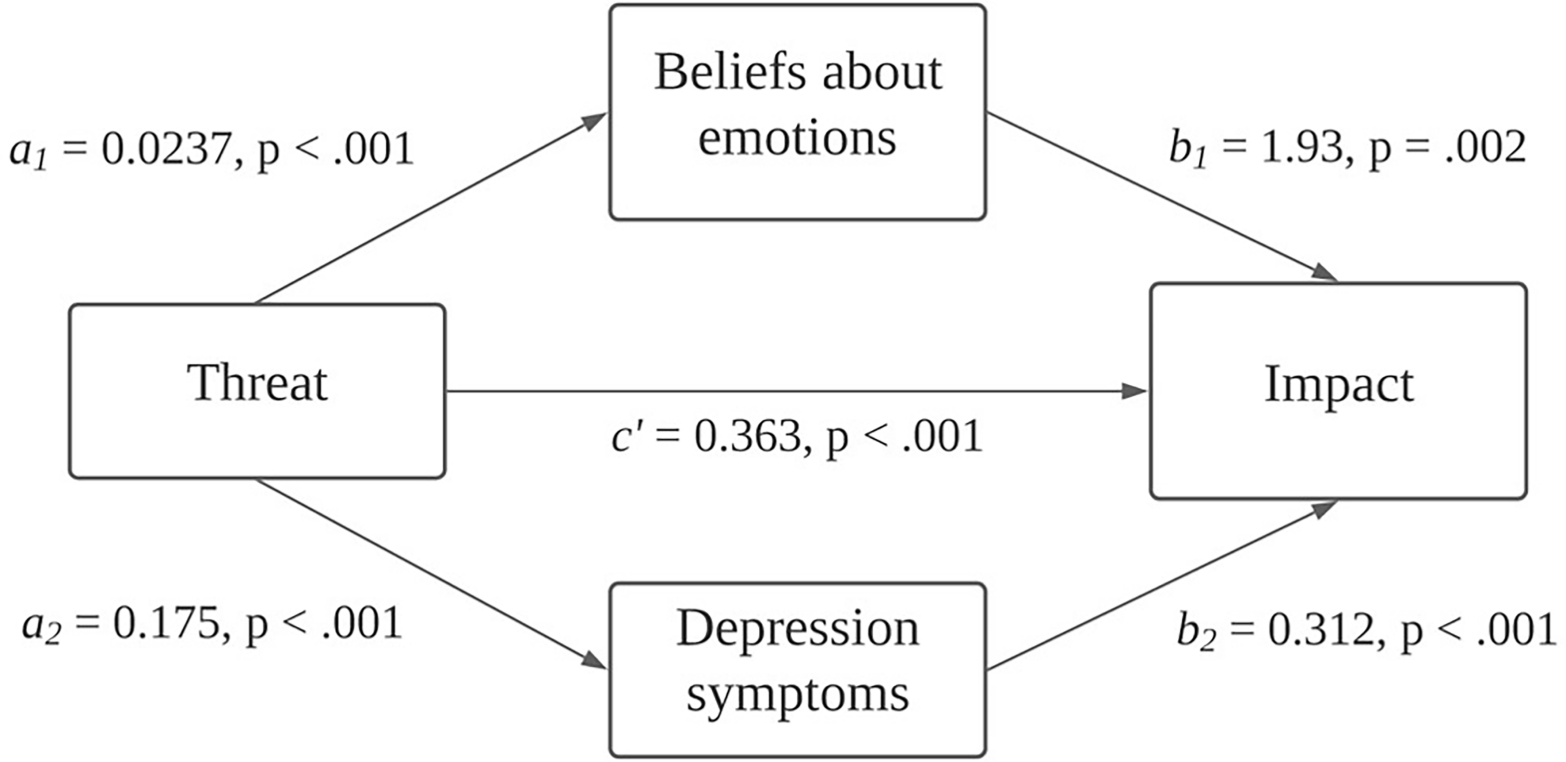
The mediating effect of depression symptoms and beliefs about emotions on emotional threat and functional impact in misophonia.

After controlling for age, gender and whether they identified with misophonia or not, beliefs about emotions and depression symptoms partially mediated the relationship between emotional threat and functional impact in misophonia (Figure 3). The indirect effect of beliefs about emotions on the relationship between threat and functional impact was 0.046, and the indirect effect of depression on threat and functional impact was 0.055, making the total indirect effect 0.100. The bootstrapped unstandardised indirect effect was 0.100, and the 95% CI ranged from 0.064 to 0.142. Both the indirect and direct effects of externalising on outbursts were statistically significant (*p* < 0.001).

## Discussion

The aim of this study was to examine two commonly reported negative outcomes of misophonia: aggressive outbursts and perceived current and future impact that misophonia has on seeing people and doing things. We aimed to explore these in relation to both misophonia-specific and more general emotional processing variables.

As hypothesised, misophonic outbursts were significantly predicted by the S-Five variable of externalising appraisals, that is, the tendency to blame others for the intensity of one’s reactions to sounds made by others. This relationship was partially explained by anxiety symptoms and disgust sensitivity. This builds on previous research finding that the relationship between misophonia and general aggressive outbursts was partly explained by symptoms of anxiety (Wu et al., 2014; Zhou et al., 2017). Neither anxiety sensitivity nor beliefs about emotions were significant independent predictor of outbursts in our exploratory regressions, which is interesting in relation to previous research finding that anxiety sensitivity strengthened the relationship between misophonia and general aggression (Schadegg et al., 2021). However, that study did not control for the presence anxiety symptoms, which our findings suggest is more relevant than fear of anxiety symptoms, for misophonia-specific outbursts, at least. Another study found that the relationship between trait neuroticism and misophonia symptoms was completely mediated by impulse control difficulties, an aspect of emotion regulation (Cassiello-Robbins et al., 2020). Theoretically, it makes sense that impulse control could be a potential key mechanism in outbursts in misophonia, and it is possible that impulse control could explain the association we found with anxiety symptoms.

Disgust sensitivity was also a significant independent predictor of misophonic outbursts. Barahmand et al. (2021) found an association between disgust sensitivity and misophonic behaviours, which included aggressive outbursts as well as non-aggressive avoidance. They found that this was completely mediated by emotional dysregulation, which we did not measure in our study. Further research is needed to clarify whether disgust sensitivity is a significant component in misophonic outbursts. If it is an important factor, then it would also be useful to examine whether this relates to core disgust in response to sounds, or socio-moral disgust (Simpson et al., 2006), which could be in response to the behaviour of the perpetrator of the sounds or directed towards oneself for having outbursts, and could have implications for treatment.

Given the cross-sectional nature of our study, the direction of these relationships between externalising appraisals, anxiety symptoms, disgust sensitivity and misophonic outbursts is not clear. One of the items in the outbursts factor relates to being afraid of doing something aggressive or violent. It is therefore possible that the association with anxiety symptoms can be accounted for by this specific item, rather than actual outbursts. It is also possible that those who have had outbursts in the past may experience more anxiety in general, in anticipation of what might happen in triggering situations or anxiety about the impact of their outbursts. It is possible that these associations could be explained by emotion dysregulation, in line with the findings of Barahmand et al. (2021) and Cassiello-Robbins et al. (2020). There may also be other variables not yet measured in misophonia research that could be contributing to these relationships, for example, a tendency to engage in angry rumination, which has previously been linked to generalised anxiety (Jessup et al., 2019), aggression and externalisation of blame (Schoenleber et al., 2021). Further exploration of this in qualitative and survey studies would be helpful for developing and testing hypotheses that may support the development of interventions.

Our second outcome variable of interest was functional impact, which was significantly predicted by the S-Five variable measuring a sense of emotional threat. This was partially explained by depression symptoms and beliefs about emotions. Previous research has also found an association between symptoms of depression and misophonia (Erfanian et al., 2019; Jager et al., 2020). Again, it is not clear whether there is a causal pathway here. It is possible that the extreme sense of dysregulation captured in the threat variable contributes to low mood, which, in turn, leads to withdrawal from activities and hopelessness about the future with misophonia. Alternatively, it could be that low mood is caused by withdrawal from activities as a result of the distress caused by misophonia. When we look at this in the context of beliefs about emotions, it is possible that beliefs that these emotions in misophonia are wrong or harmful could also lead to withdrawal from other people. The LESS II captures a wide range of negative beliefs about emotions, with a broader theme of emotions being bad, wrong or harmful, as opposed to fear of emotions as captured in the measures of anxiety and disgust sensitivity. Therefore, negative beliefs about emotions might contribute to functional impact through behavioural responses to shame or guilt about emotions in misophonia, as opposed to a fear of emotions captured in the sensitivity measures (which were not significant independent predictors). There could also be another variable or set of variables that could further explain these relationships. Along these lines, the tendency to ruminate in a depressive way could be associated with greater sense of threat, low mood, more negative beliefs about emotions and greater avoidance, withdrawal and hopelessness. In-depth interviews and prospective and experimental studies are needed to shed light on these relationships.

It was interesting to find that interoception was not significantly correlated with any of our S-Five outcome or predictor variables in the group who identified with having misophonia. This was surprising considering previously found associations between interoception and misophonia symptoms (Kumar et al., 2017; McKay et al., 2018). Examining our correlations further, we noted that interoception had low, significant correlations with overall misophonia severity and with the internalising appraisals factor, suggesting that interoception might play a part in some aspects of misophonia but not others. We also found that it was significantly (albeit weakly) correlated to all S-Five variables in the group that did not identify with misophonia. It would be interesting to test this association further in future research, perhaps investigating whether one’s misophonia “status” moderates the relationship between interoception and misophonia symptoms.

### Limitations

There were some limitations to this study. Most importantly, this was an exploratory study, and our results should therefore be considered theories to be tested further. We recruited from two different populations and used self-identification with misophonia (or not) to create our initial two groups. We then combined the sample for our regression analyses, controlling for the “identification with misophonia” variable in all regression analyses. While there are currently no published cut-off scores on the S-Five, we note that the mean score for the S-Five total in our sample of those identifying with misophonia (mean = 147.15, data available on request) was comparable to the means presented in the original validation of the S-Five in a sample of individuals identifying with the condition (mean = 148.0). Future studies testing theories emerging from this research would benefit from using either community or clinical samples, with a view to later doing comparison studies between clinical and non-clinical groups, using gold standard diagnostic interviews to create clinical groups. However, this is difficult to achieve without agreed diagnostic criteria for misophonia. Additionally, participants recruited from misophonia social media groups were disproportionately female, consistent with other research on misophonia. Future studies would benefit from a more balanced sample and testing for differences between gender groups. Finally, we used the combined total of the Disgust Sensitivity and Propensity scale as our measure of disgust sensitivity. In future studies, it would be preferable to separate these two constructs and test whether both have a direct impact on aspects of misophonia.

### Summary and Conclusion

This exploratory study is, to our knowledge, the first to investigate potential predictors of two misophonia-specific outcomes: outbursts and functional impact. Our findings suggest that these two aspects of misophonia are related to cognitive and emotion processes, both misophonia-specific and non-specific. This highlights the importance of breaking misophonia down into its different dimensions to improve our understanding of the condition and its consequences. The study provides further support for the notion that there are psychological aspects to misophonia, which raises hope for developing and adapting psychological interventions to improve the lives of those suffering with the condition.

## Data Availability Statement

The raw data supporting the conclusions of this article will be made available by the authors, without undue reservation.

## Ethics Statement

The studies involving human participants were reviewed and approved by the King’s College London Psychiatry, Nursing and Midwifery Research Ethics Subcommittee (REC reference number: HR-19/20-17173). The patients/participants provided their written informed consent to participate in this study.

## Author Contributions

QW completed the analysis, contributed to interpretation, and contributed to the manuscript. SV provided supervision to the project, carried out data collection, contributed to data analysis, and contributed to the manuscript (Materials and Methods and Results). NU-M carried out data collection and contributed to data analysis. JG provided supervision to the project, contributed to interpretation, and contributed to the manuscript. All authors contributed to the article and approved the submitted version.

## Funding

SV was funded or partially funded by the Biomedical Research Centre for Mental Health at South London and Maudsley NHS Foundation Trust and King’s College London. This research was funded in whole, or in part, by the Wellcome Trust (JG; grant number 102176/B/13/Z).

## Author Disclaimer

The views expressed are those of the author(s) and not necessarily those of the NHS, Wellcome Trust, the NIHR or the Department of Health and Social Care.

## Conflict of Interest

The authors declare that the research was conducted in the absence of any commercial or financial relationships that could be construed as a potential conflict of interest.

## Publisher’s Note

All claims expressed in this article are solely those of the authors and do not necessarily represent those of their affiliated organizations, or those of the publisher, the editors and the reviewers. Any product that may be evaluated in this article, or claim that may be made by its manufacturer, is not guaranteed or endorsed by the publisher.

## Footnotes

^1^Leahy, R. L. (2012). Leahy Emotional Schema Scale II (LESS II). American Institute for Cognitive Therapy (Unpublished Manuscript).
